# Supplementary material for: Isolation and Characterization of Strain Exiguobacterium sp. KRL4, a Producer of Bioactive Secondary Metabolites from a Tibetan Glacier
Source: Microorganisms. 2021 Apr 21;9(5):890. doi: 10.3390/microorganisms9050890 (PMC8143284; doi:10.3390/microorganisms9050890)
Supplement: Supplementary file 1 [file microorganisms-09-00890-s001.zip › microorganisms-1186397-supplementary.pdf]

Table S1. Project information.

MIGS ID	PROPERTY	TERM
**MIGS 31**	Finishing quality	Improved-high-quality-draft
**MIGS-28**	Libraries used	Illumina paired-end library
**MIGS 29**	Sequencing platforms	Illumina MiSeq
**MIGS 31.2**	Fold coverage	114x
**MIGS 30**	Assemblers	CLC Genomics workbench v 7.5.1
**MIGS 32**	Gene calling method	RAST/PGAP
	Locus Tag	BLD48
	Genbank ID	MOLV00000000.1
	GenBank Date of Release	07-Nov-2016
	GOLD ID	NOT REGISTERED
	BIOPROJECT	PRJNA350615
**MIGS 13**	Source Material Identifier	
	Project relevance	Pharmaceutical

Table S2. Peaks list of FAMEs from GC-MS analysis with relative abundance indicated.

Peak #	*Start RT*	*End RT*	*Area*	*%Area*
1	7.65	7.87	3.051.800.287	2.39
2	9.28	9.44	2.478.062.528	1.94
3	9.50	9.60	2.076.911.907	1.62
4	9.62	9.73	7.944.850.288	6.21
5	9.96	10.10	7.515.367.087	5.88
6	10.59	10.75	4.260.692.434	3.33
7	11.07	11.22	24.307.015.339	19.00
8	11.24	11.33	2.889.259.050	2.26
9	11.34	11.44	2.398.989.121	1.88
10	13.04	13.16	50.216.135.074	39.26
11	13.17	13.25	2.487.499.744	1.94
12	13.51	13.62	8.038.254.542	6.28
13	13.85	13.96	637.560.014	0.50
14	14.60	14.69	627.607.671	0.49
15	16.36	16.50	1.995.372.092	1.56
16	16.54	16.70	2.862.982.667	2.24
17	23.30	23.42	4.123.673.734	3.22

Table S3. Number of genes protein coding genes of *Exiguobacterium sp*. KRL4 associated with general COG functional categories.

CODE	VALUE	%AGE	DESCRIPTION
**J**	150	4.6	Translation, ribosomal structure and biogenesis
**A**	0	0	RNA processing and modification
**K**	162	5	Transcription
**L**	162	5	Replication, recombination and repair
**B**	0	0	Chromatin structure and dynamics
**D**	23	0.7	Cell cycle control, Cell division, chromosome partitioning
**V**	0	0	Defense mechanisms
**T**	127	3.7	Signal transduction mechanisms
**M**	120	3.9	Cell wall/membrane biogenesis
**N**	58	1.8	Cell motility
**U**	0	0	Intracellular trafficking and secretion
**O**	81	2.5	Posttranslational modification, protein turnover, chaperones
**C**	124	3.8	Energy production and conversion
**G**	162	5	Carbohydrate transport and metabolism
**E**	179	5.5	Amino acid transport and metabolism
**F**	71	2.2	Nucleotide transport and metabolism
**H**	78	2.4	Coenzyme transport and metabolism
**I**	84	2.6	Lipid transport and metabolism
**P**	117	3.6	Inorganic ion transport and metabolism
**Q**	26	0.8	Secondary metabolites biosynthesis, transport and catabolism
**R**	250	7.7	General function prediction only
**S**	165	5.1	Function unknown
**-**	1008	31.8	Not in COGs

The total is based on the total number of protein coding genes in the genome.

Table S4. List of genes identified in the terpenes and siderophore pathways.

GENE	PUTATIVE FUNCTION
**CLUSTER 1: TERPENE**	
**CTG1_41**	Class I SAM-dependent methyltransferase
**CTG1_42**	Phytoene desaturase
**CTG1_43**	Phytoene desaturase
**CTG1_44**	Phytoene synthase
**CTG1_45**	Phytoene desaturase
**CTG1_46**	Glycosyl transferase
**CTG1_47**	Glycerol acyltransferase
**CTG1_52**	enoil-CoA hydratase
**CTG1_54**	alpha glycosidase
**CLUSTER 2: TERPENE**	
**CTG1_1452**	Phytoene synthase
**CTG1_1453**	alpha-beta hydrolases
**CTG1_1455**	diguanilate cyclase
**CTG1_1460**	N-acetyltransferase
**CLUSTER 3: SIDEROPHORE**	
**CTG1_1637**	iucA/iucC siderophore synthesis
**CTG1_1638**	iucA/iucC siderophore synthesis
**CTG1_1639**	acyl-CoA synthase
**CTG1_1640**	putative acyl carrier protein
**CTG1_1643**	glycerol dehydrogenase

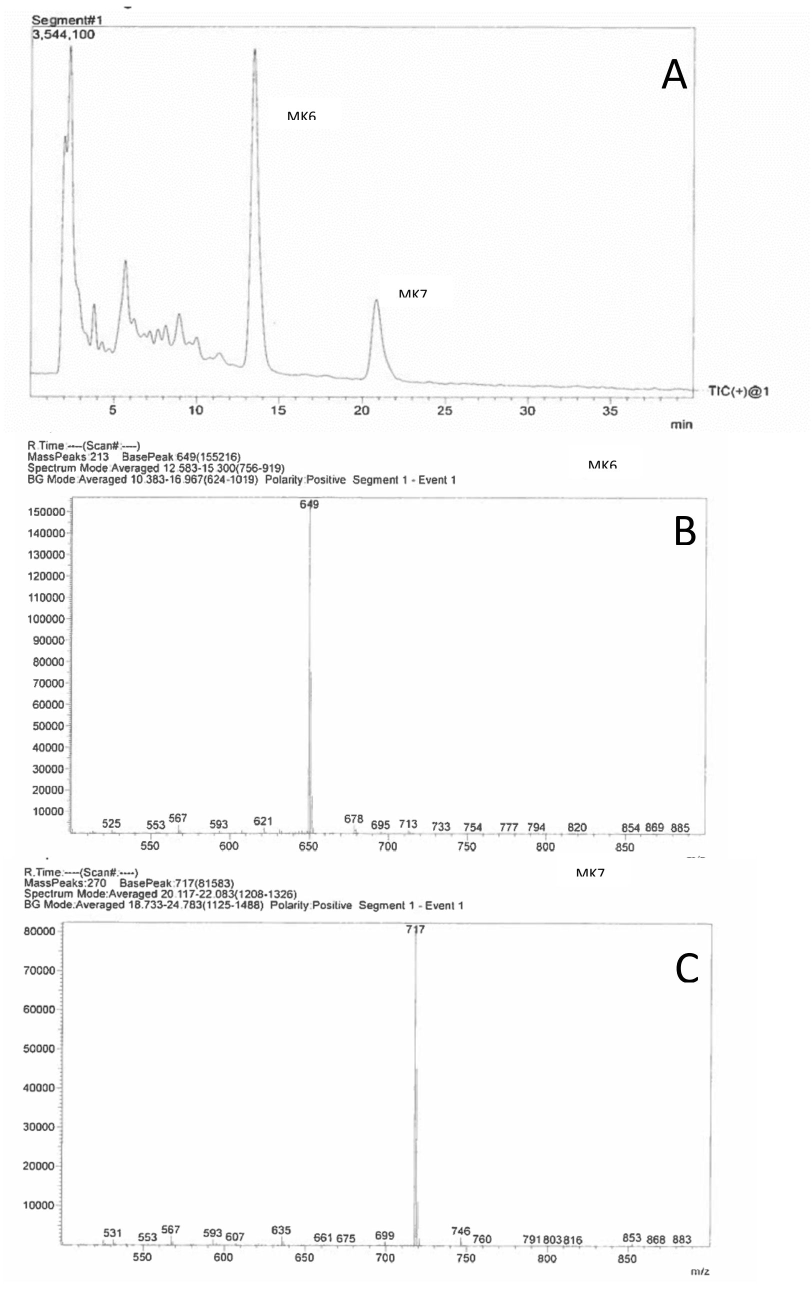

Figure S1.LC-MS analysis of quinones. A) Chromatogram of extracted quinones analysed on a reverse-phase RP-18 Lichrospher column eluted with n-hexane/ethylacetate (99:1, by vol.) with a flow rate of 1.0 mL min-1. Identification by ESI/MS of quinone MK6 (B) and quinone MK7 (C).

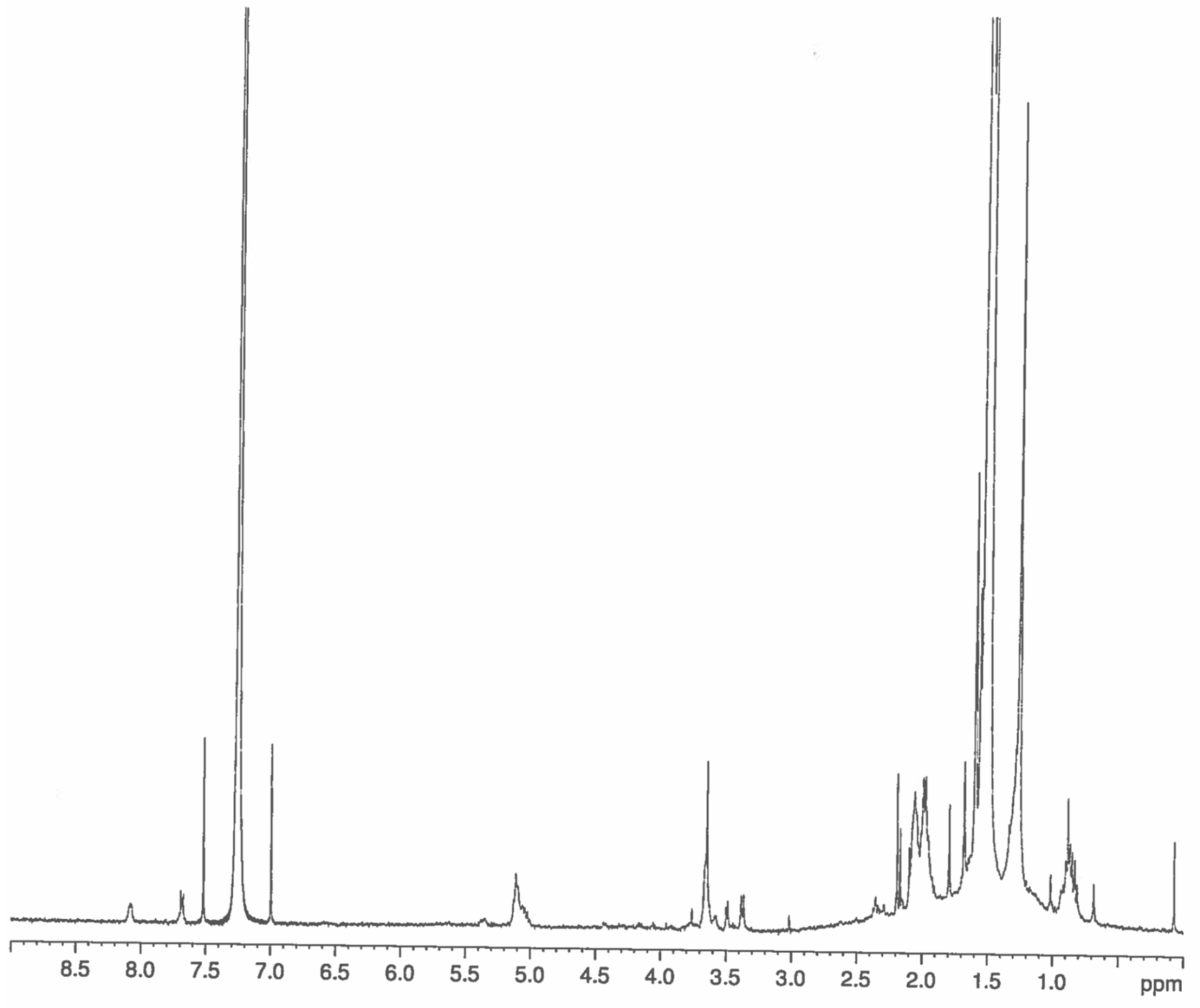

Figure S2. ^{1}H-NMR spectrum of extracted quinones.

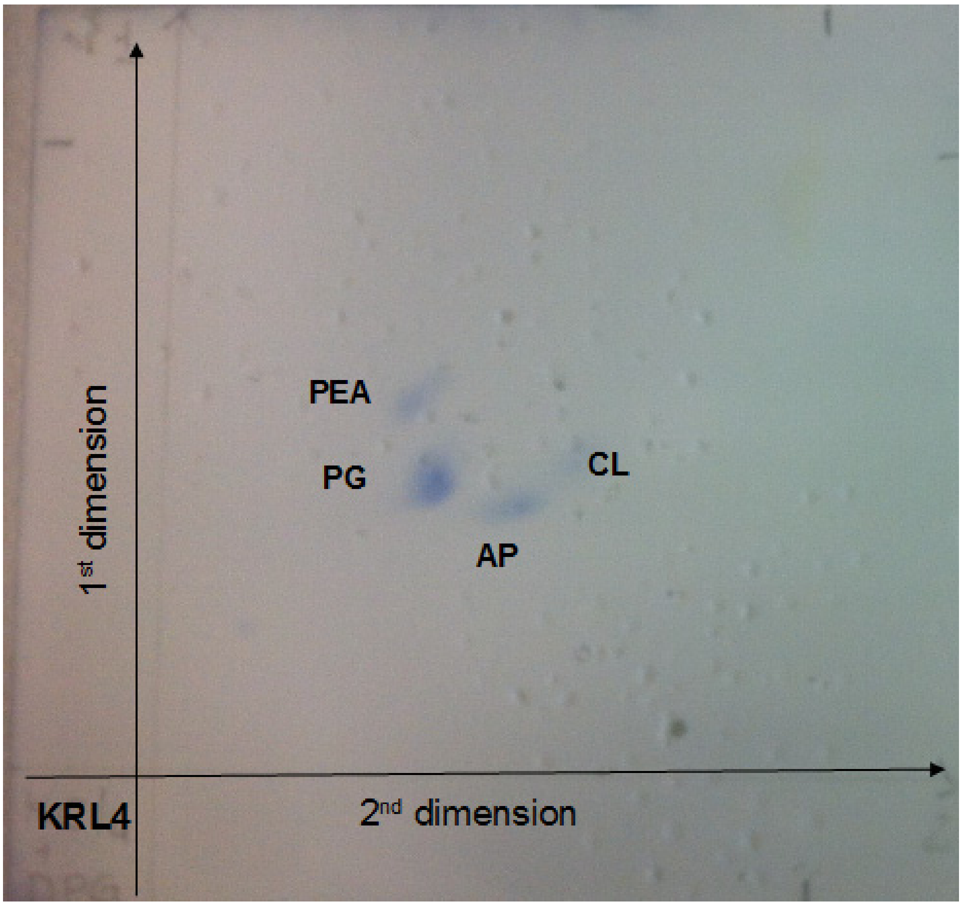

Figure S3. Bi-dimensional Thin Layer Chromatography (TLC) showing the phospholipids of strain KRL4. First dimension: $CHCl_3$/MeOH/H_2O (65:25:4, by vol.); second dimension: $CHCl_3$/MeOH/Acetic acid/H_2O (80:12:15:4, by vol.). Phospholipids were detected by spraying TLC with the Dittmer- Lester reagent. 1,2 diacylglycero-3-phosphorylethanolamine (PEA), 1,2 dipalmitoyl-3-glycero-phosphatidic acid (AP), 1-(3-sn-phosphatidyl)-sn-glycerol disodium salt (PG), 1,3-di(3-sn-phosphatidyl)-sn-glycerol disodium salt (cardiolipin, CL).

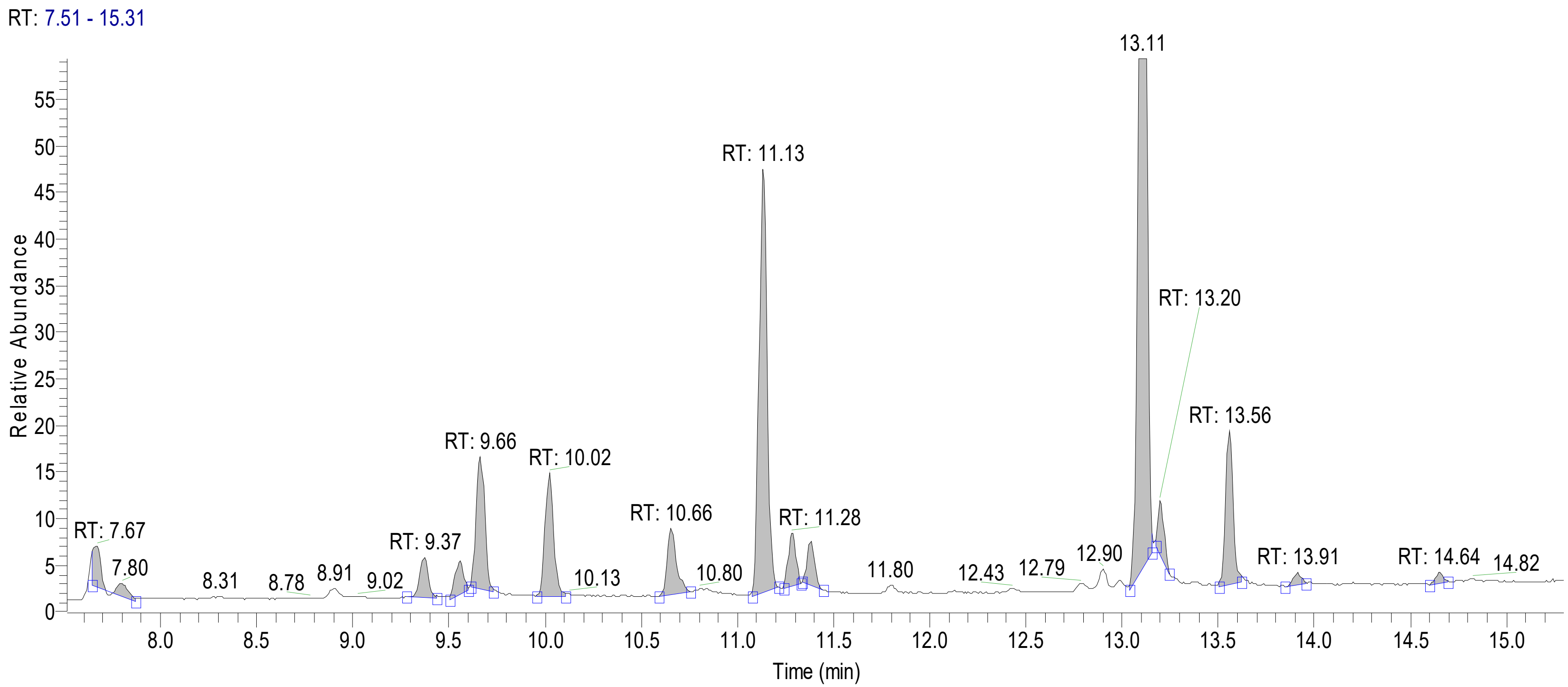

Figure S4. Total Ion Chromatogram of FAMEs obtained by GC-MS analysis.

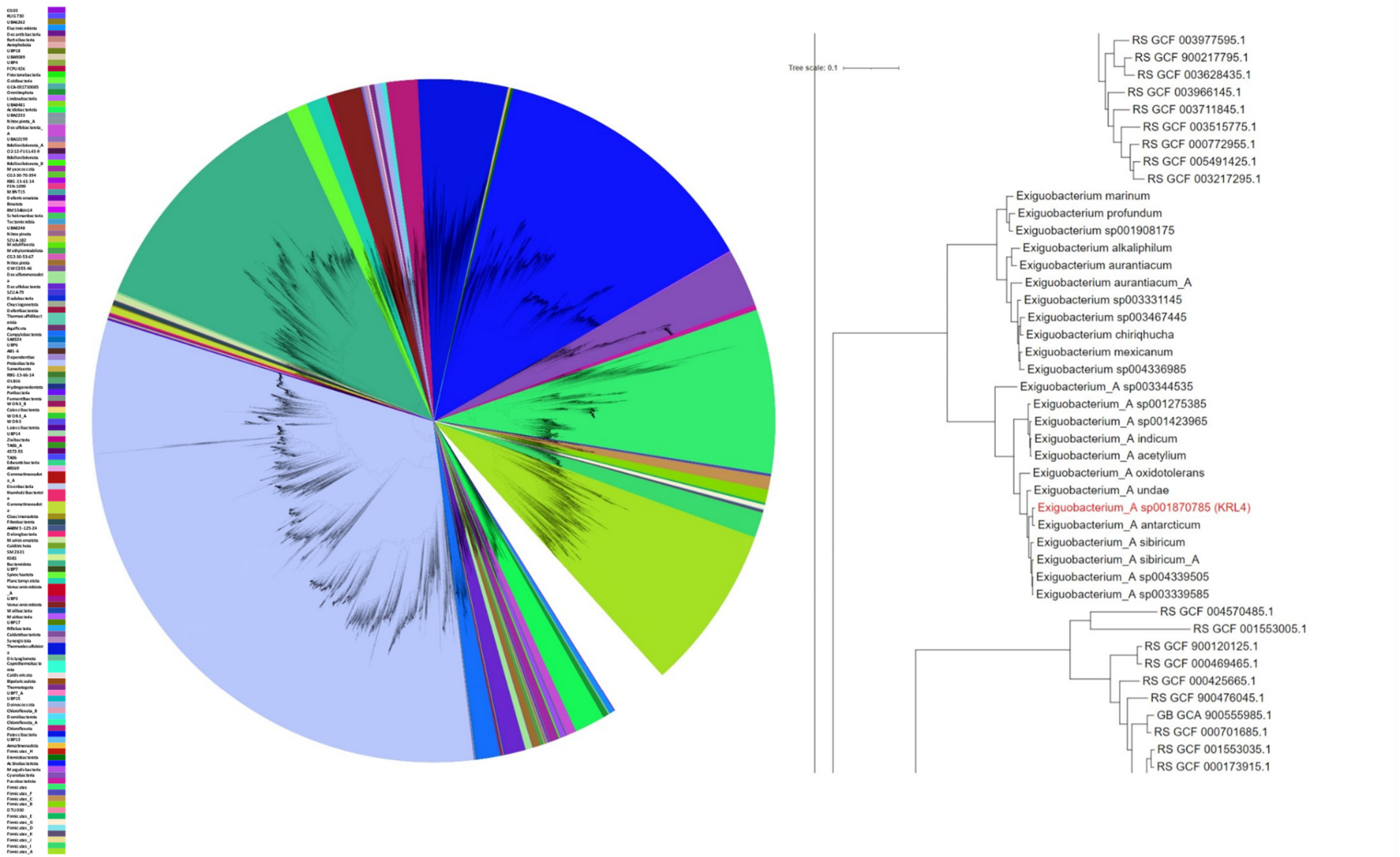

Figure S5. Whole genome-based classification of strain KRL4 according the GTDB bacterial phylogeny
